# OncoVee™-MiniPDX-guided anticancer treatment for HER2-negative intermediate-advanced gastric cancer patients: a single-arm, open-label phase I clinical study

**DOI:** 10.1007/s12672-023-00661-y

**Published:** 2023-04-24

**Authors:** Baonan Zhang, Yuzhen Li, Xiaodan Zhu, Zhe Chen, Xiaona Huang, Tingjie Gong, Weiwang Zheng, Zhenle Bi, Chenyang Zhu, Jingyi Qian, Xiaoqiang Li, Chunhui Jin

**Affiliations:** 1 Department of Oncology, Wuxi Hospital Affiliated to Nanjing University of Chinese Medicine, 8 West Zhongnan Road, Wuxi, 214071 China; 2 Department of Medical, Co. Ltd. Shanghai, Shanghai LIDE Biotech, China

**Keywords:** Mini patient-derived xenograft (MiniPDX) animal model, Gastric cancer, Progression-free survival (PFS), Survival time, Response

## Abstract

**Background:**

Chemotherapy is the main treatment strategy for patients with advanced HER2-negative gastric cancer (GC); yet, many patients do not respond well to treatment. This study evaluated the sensitivity of a mini patient-derived xenograft (MiniPDX) animal model in patients with HER2-negative intermediate-advanced GC.

**Methods:**

In this single-arm, open-label clinical study, we consecutively recruited patients with HER2-negative advanced or recurrent GC from September 2018 to July 2021. Tumor tissues were subjected to MiniPDX drug sensitivity tests for screening individualized anti-tumor drugs; appropriate drug types or combinations were selected based on drug screening results. The primary endpoints were progression-free survival (PFS) and safety, and the secondary endpoints were overall survival (OS) and objective response rate (ORR).

**Results:**

A total of 17 patients were screened, and 14 eligible patients were included.The median follow-up time was 9 (2–34) months. The median PFS time was 14.1 (2–34) months, the median OS time was 16.9 (2–34) months, ORR was 42.9% (6/14), and DCR was 92.9% (13/14). The most common treatment-related adverse events (TRAE) were fatigue (14 (100%)), anorexia (13 (93%)) and insomnia (12 (86%)), and the most common grade 3 or worse TRAE was fatigue (6 (43%)), and anorexia (6 (43%)). The occurrence rate of myelosuppression, nausea and vomiting, abnormal liver enzymes, and other grade 3–4 chemotherapy adverse reactions were relatively low, and no grade 5 treatment-related adverse events occurred.

**Conclusion:**

Screening HER2-negative medium-advanced GC/GJC chemotherapy regimens and targeted drugs based on MiniPDX animal models showed good tumor activity and safety.

**Supplementary Information:**

The online version contains supplementary material available at 10.1007/s12672-023-00661-y.

## Introduction

According to the latest global cancer burden data recently released by the International Agency for Research on Cancer (IARC) [1], 1.1 million people were diagnosed with gastric cancer (GC) in 2020. Gastric cancer has the highest morbidity and mortality rate among malignant digestive system tumors. Most cases are diagnosed with an advanced stage. Among advanced-stage GC patients who receive surgery, 60% experience local recurrence or distant metastasis. Human epidermal growth factor receptor 2 (HER2) is a member of the receptor family related to tumor cell proliferation, apoptosis, adhesion, migration and differentiation, and is involved in the pathogenesis and adverse results of a variety of cancers, including advanced gastric cancer (AGC) and gastroesophageal junction cancer (GJC) [2, 3]. The positive rate of HER2 in GC was about 12% ~ 20% [4]. Trastuzumab is a monoclonal antibody targeting HER2, which exerts antitumor effects by inducing antiantibody dependent cytotoxicity and inhibiting HER2-mediated signalling [5]. The phase 3 ToGA study demonstrated the clinical benefits of trastuzumab combined with chemotherapy in patients with previously untreated, unresectable or metastatic HER2-positive (HER2 +) GC or GEJ cancer [6], and HER2-targeted combined chemotherapy has become the first-line treatment for HER2-positive advanced GC [7, 8]. Yet, there are no standard first-line treatments for advanced or recurrent gastric cancer with HER2 negative. Immunotherapy combined with chemotherapy is a common treatment approach; still, a high number of patients do not respond well to treatment. Monotherapy is the most common second- and third-line treatment [9, 10]. Accordingly, Precision treatment is particularly important for patients with HER2-negative advanced or recurrent gastric cancer.

The patient-derived xenograft (PDX) model has come into our view, which is commonly used for in vivo experiments. Compared to animal models established using immortalized cell lines, the use of patient tissue can more realistically reflect the molecular diversity and cellular and histological heterogeneity of tumors in patients. PDX model has been widely used for drug evaluation, biomarker identification, biological research, and personalized medicine strategies [11–13]. However, the establishment of PDX may require a longer time (4 to 8 months) compared to other models, which is also a major limitation considering that most patients with advanced-stage have a poor prognosis. Thus, a MiniPDX model has been proposed. MiniPDX model is established by injecting patient-derived tumor cells into hollow fiber capsules, which significantly saves time [14]. So far, this model has been applied to a variety of solid tumors [15].

The application of miniPDX model in the treatment of GC has also been reported [15]. Zhu et al*.* [16] found that drugs screened by OncoVeeTM-Mini-PDX have significant benefits for a patient with HER2-positive advanced gastric cancer. Moreover, Wang et al*.* [17] successfully established a MiniPDX model for four patients (three patients were HER2 positive, and one patient was HER2 negative), where two patients achieved partial remission after treatment, while the disease progressed in the other two patients, and no serious adverse reactions were observed. In addition, Ge et al*.* found that MiniPDX can prolong the survival of patients with HER2-negative gastric cancer with liver metastases (GCLM) and improve efficacy [18]. In this study, we established an individualized drug screening system through the MiniPDX model, and used a prospective single-arm open-label trial aiming to prolong the survival of HER2-negative GC patients.

## Materials and methods

### Selection criteria of patients

This single-arm clinical study (registration number: ChiCTR1800019568; registration institution: Chinese Clinical Trial Registry) evaluated the safety and antitumor activity of an individualized drug screening protocol established by the MiniPDX model in patients with advanced GC/GJC.

Inclusion criteria:1.GC/GJC patients aged between 18 and 80 who cannot undergo surgery.2.It is a stage III/IV intermediate-advanced malignancy confirmed by imaging or histology, and is negative for Her-2 as confirmed by FISH.3.The time from the start of the last chemotherapy is ≥ 4 weeks.4.Fresh samples of tumor tissue can be obtained by surgical excision or biopsy 5. At least one objective tumor that meets the RECIST 1.1 standard can be evaluated using CT/MRI for efficacy 6. The ECOG score is ≤ 2 points, and the expected survival period must be ≥ 3 months.

Exclusion criteria: 1. Patients who cannot obtain sufficient tumor cells.2. Patients who cannot receive follow-up or are participating in other clinical trials. 3. Patients with severe cardiovascular and cerebrovascular diseases and severe abnormalities in liver and kidney functions. 4. Patients with multiple cancers.

Exclusion criteria: 1. Subject withdraws informed consent 2. Those who have lost follow-up within half a year after enrollment and have not been followed up as required. 3. The researcher believes that the subject is not suitable for continuing to participate in the trial.

### OncoVeeM-MiniPDX model establishment and drug sensitivity test

The MiniPDX model was established by taking the primary tumor specimens resected by gastroscopic biopsy or puncture or palliative surgery. Drug susceptibility was detected by the OncoVee™ MiniPDX method (Shanghai LIDE Biotech Co., Ltd.)[14]. GC tissue samples were washed with Hank’s balanced salt solution (HBSS) to remove non-tumor tissues, such as blood vessels and necrotic tumor tissues. After sectioning, the tumor tissues were digested with collagenase at 37 °C for 1–2 h, the cells were collected, and blood cells and fibroblasts were removed. The GC cell suspension was then transferred to HBSS rinsed capsules.

BALB/c nude mice with 4-week-old (SLARC Inc., Shanghai, China) were selected as animal models. A total of 4–6 capsules were implanted subcutaneously on their backs. One day after inoculation of tumor cells, according to the treatment plan given by the researcher, appropriate drugs or their combinations (Table 1) were given to mice for 7 days, and nude mice treated with normal saline were set up as controls. After 7 days, the cells in the capsules were taken out, and the relative fluorescence units (RFU) of tumor cells were measured by CellTiter-Glo fluorescence cell viability assay. The relative proliferation rate of tumor cells was calculated (T/C%) (T/C% = (RFU of drug treatment group on day 7 − RFU of drug treatment group on day 0) / (RFU of the control group on day 7 − RFU of the control group on day 0) × 100%)). Each experiment was conducted six times and the average value was reported. When the tumor growth inhibition rate was > 50% compared with the blank group, the regimen was defined “sensitive” [20].

**Table 1:** Drug preparations and treatment details

Drug	Dose	Usage^a^
AZD9291	5 mg/kg	PO, QD*7
Anlotinib	3 mg/kg	PO, QD*7
Apatinib	100 mg/kg 40 mg/kg	PO, QD*7 PO, QD*7
S-1	10 mg/kg 5 mg/Kg	PO, QD*5 PO, QD*5
Docetaxel	20 mg/kg	IP, Q4D*2
Capecitabine	400 mg/kg	PO, QD*7
Irinotecan	50 mg/kg	IP, Q4D*2
Oxaliplatin	5 mg/kg	IP, QW
Paclitaxel	20 mg/kg 20 mg/kg	IP, QW IP, Q4D*2
5-FU	15 mg/Kg	IP, QD*5
CF	50 mg/Kg	IP, QW
Cisplatin	5 mg/kg	IP, QW
Epirubicin	5 mg/kg	IP, QW

^a^Dosing route, dosing frequency followed by, where indicated, dosing times and/or treatment duration;po oral, ip intraperitoneal, qd once a day, qw once a week, q4d once every 4 days

The chemotherapy regimen with the highest chemosensitivity was given to the patients corresponding to the tumor cells for chemotherapy, and the drug was used until disease progression, death, intolerance of adverse reactions, withdrawal of consent, investigator’s decision, or completion of the 24-month study.

### Results and measurements

The primary endpoints of this study were progression-free survival (PFS) and safety. Secondary endpoints were overall survival (OS), objective response rate (ORR), and disease control rate (DCR), all determined according to RECIST 1.1. PFS was defined as the time from the first drug administration to the first documented disease progression or death from any cause, whichever occurred first. OS was defined as the time from first drug administration to death from any cause. Patients were divided into four subgroups: complete remission (CR), partial remission (PR), stable disease (SD), and progressive disease (PD). ORR was defined as the percentage of CR and PR patients among all patients. DCR was defined as the percentage of patients achieving CR, PR, and SD.

All patients underwent routine CT scanning of the upper abdomen during baseline and follow-up using a Somatom PLUS-S CT scanner (Siemens Medical Systems, Erlangen, Germany). CT images were processed using the 3D slice software package (version 4.7). At least two radiologists with 10 + years of work experience and a research assistant completed the process. During treatment, radiographic assessments of short-term efficacy were performed every two cycles until disease progression or death, according to RECIST 1.1. Adverse events were monitored and graded using the National Cancer Institute Common Terminology Criteria for Adverse Events (version 4.0), and all adverse events were reported from the time of treatment assignment to 90 days after treatment cessation.

### Follow up

Follow-up was performed every 6 weeks during treatment and every 3 months after treatment until death. Follow-up evaluation included medical history, physical examination, blood routine, liver and kidney function, and related tumor markers, with telephone follow-up for those who were not regularly reviewed.During each follow-up, patients’ quality of life was quantitatively measured by the QLQ-C30 scale, from overall health, functional dimensions (including physical function, role function, emotional function, cognitive function, and social function), and symptom-related dimensions (including fatigue, nausea, and vomiting, pain, dyspnea, insomnia, poor appetite, constipation, diarrhea, etc.) to evaluate and calculate the standard score (SS) of the overall health status: *SS* = [(RS-1)/6] *100, RS = *(Overall health status score* + *Overall quality of life score)/2.*

### Statistical analysis

Microsoft Excel 2019was used to establish a database, SPSS 22.0 was used for statistical analysis, and GraphPad Prism 8.0was used for plotting figures. The Kolmogorov-Smirnov (K-S) method and the Shapiro–Wilk (S-W) method were applied for the normality test. The age distribution of the patients conformed to the normal distribution and was expressed asX ± SD. The PFS and OS curves were calculated by the Kaplan-Meier method. The overall quality of life scores of patients before and after treatment were compared using paired T-test. P < 0.05 was considered to be statistically significant.

### Ethical approval

This study was approved by the Ethics Committee of Wuxi Hospital Affiliated to Nanjing University of Chinese Medicine (201809001J01-01). The animal study was reviewed and approved by the Institutional Animal care and use Committee(IACUC) Shanghai LIDI Biotech,Co.LTD (LDIACUC001). This research study was conducted following the Declaration of Helsinki, and all patients signed informed consent.All institutional and national guidelines for the care and use of laboratory animals were followed.

## Results

### General information of patients

**Fig. 1:**
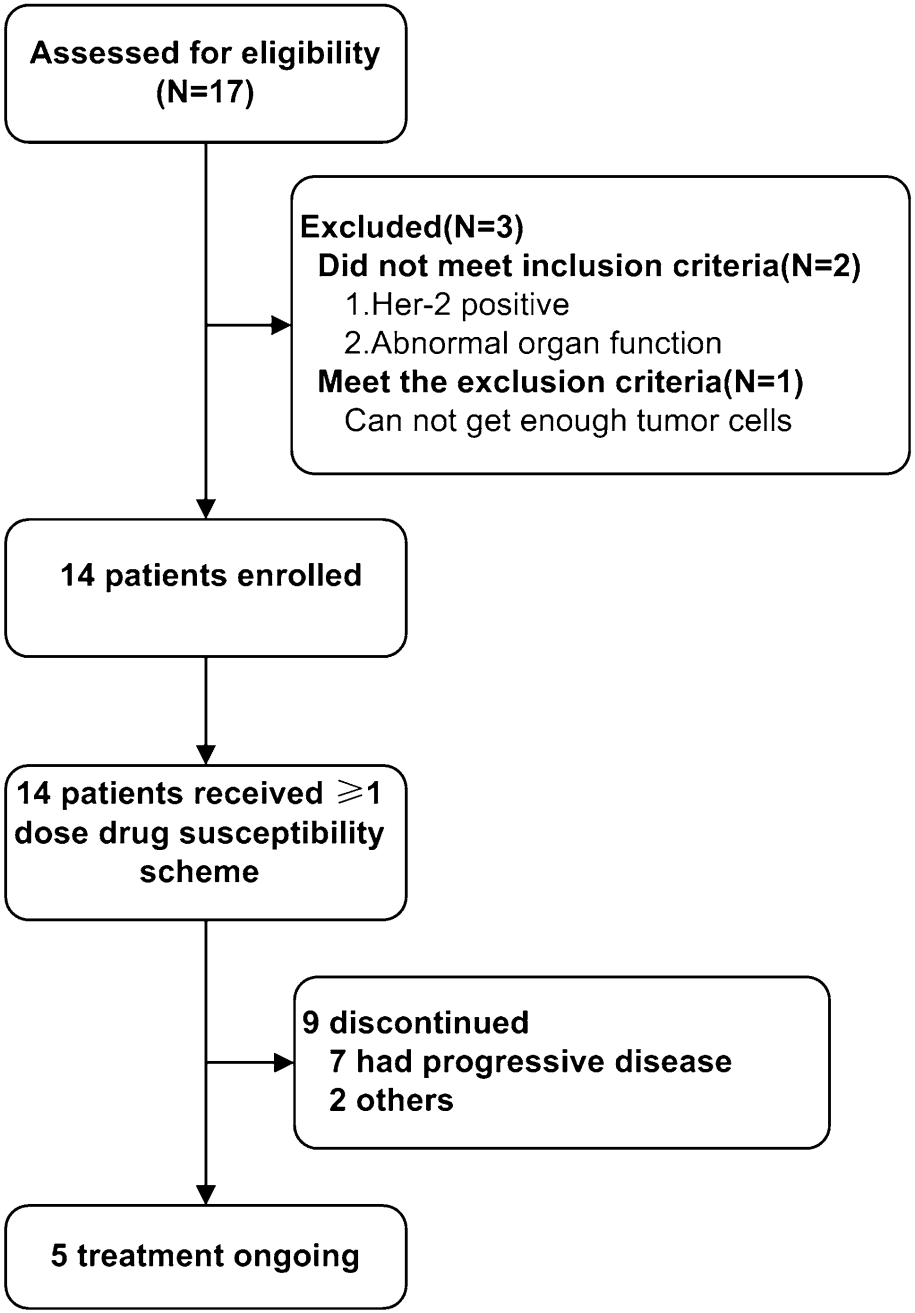
Flow chart of the study

Between September 2018 and July 2021, 17 patients were screened; 2 patients did not meet the inclusion criteria and 1 patient met the exclusion criteria (Fig. 1). The MiniPDX model was successfully established in the remaining 14 patients, and at least one course of drug susceptibility testing was included in the primary analysis. The mean age of the 14 patients was 71.57 ± 9.78 years. Eleven (79%) patients had GC; 3 (21%) patients had GJC. In terms of clinical-stage, 10 (71%) patients were at stage IV, 3 (21%) patients were at stage IIIC, and 1 (7.14%) was at stage IIIB. In addition, 4 (29%) patients had one distant metastasis, 5 (36%) patients had multiple distant metastases, and the others had no distant metastases.Five patients had no previous treatment, seven patients had first-line treatment, and two patients had second-line or third-line treatment. (Table 2). The median follow-up time for data analysis (data cutoff was December 31, 2021) was 9.0 (2–34) months. The most common reason for discontinuation of treatment was disease progression in 7 (50%) patients and adverse events in 2 (14%) patients (Fig. 1).

**Table 2:** Baseline characteristics of patients

Number	Age	Gender	Tumor stage*	Part	TNM-stage^a^	Number of previous treatment lines	Metastasis locations	Pathological types
1	69	male	IV	GJC	T3N0M1	1	Single	Adenocarcinoma
2	75	male	III	GJC	T3N2M0	0	None	Adenocarcinoma
3	64	male	III	GC	T4aN1M0	0	None	Adenocarcinoma
4	86	male	IV	GC	TxN1M1	1	Multiple	Adenocarcinoma
5	79	male	III	GC	T4bN1M0	0	None	Adenocarcinoma
6	66	male	IV	GC	TxN1M1	2	Multiple	Adenocarcinoma
7	64	male	IV	GC	TxN1M1	1	Multiple	Adenocarcinoma
8	82	male	IV	GC	TxN1M1	0	Single	Adenocarcinoma
9	69	male	IV	GC	TxN1M1	1	Multiple	Adenocarcinoma
10	69	female	IV	GC	TxN1M1	1	Multiple	Adenocarcinoma
11	50	female	IV	GC	TxN0M1	1	Single	Signet ring cell carcinoma
12	82	male	IV	GC	TxN0M1	0	Single	Adenocarcinoma
13	81	male	III	GJC	T1N0M0	3	None	Adenocarcinoma
14	66	male	IV	GC	T3N2M1	1	None	Adenocarcinoma

^a^Tumor stage and TNM-stage were defined according to the American Joint Committee on Cancer (AJCC)TNM staging system (AJCC 8th edition)

### Results of MiniPDX model detection

The intraoperative tumor tissues of 14 patients in the drug-susceptibility group were submitted for examination, and a MiniPDX model was established. According to the pathological type of the patient’s tumor and the basic condition of the patient, the researchers proposed 3 treatment regimens in combination with the NCCN guidelines (2016 edition) [19], which were then applied to the mice for drug susceptibility testing. The results showed that an average of 0.5 (± 0.31) different types of regimens showed a satisfactory inhibitory effect (T/C% < 50%) for each patient (Table 3).

**Table 3:** Mini-PDX Drug Sensitivity Results for the 14 Patients

Number	Chemotherapy regimen^a^	Inhibition rate (1-T/C%)^b^	Sensitive^c^
1	Apatinib	59%	+
	AZD9291	46%	−
	Anlotinib	40%	−
2	Irino + S-1	84%	+
	Doc + S-1	71%	+
	Cape + Oxa	60%	+
3	Oxa + S-1	84%	+
	Doc + S-1	71%	+
	Irino + S-1	35%	−
4	S-1 + Pac	86%	+
	S-1 + Oxa	53%	+
	S-1 + Irino	49%	−
5	S-1 + Doc	51%	+
	S-1 + Oxa	29%	−
	S-1 + Irino	11%	−
6	S-1 + Oxa	14%	−
	Doc	-11%	−
	Irino	-17%	−
7	S-1 + Oxa	66%	+
	S-1 + Doc	64%	+
	S-1 + Irino	27%	−
8	S-1 + Oxa	60%	+
	S-1 + Apa	26%	−
	Apa + Pac	13%	−
9	Pac + S-1 + Apa	68%	+
	Irino + Cape + Apa	53%	+
	Oxa + Cape + Apa	43%	−
10	Cis + Cape + Apa	75%	+
	Pac + S-1 + Apa	61%	+
	Irino + 5-FU + CF + Apa	44%	−
11	Epi + Cis + 5-FU + Apa	88%	+
	Cis + Cape + Apa	84%	+
	Irino + 5-FU + CF + Apa	79%	+
12	S-1 + Oxa + Apa	75%	+
	S-1 + Pac + Apa	46%	−
	S-1 + Irino + Apa	33%	−
13	S-1 + Oxa + Apa	64%	+
	S-1 + Irino + Apa	41%	−
	S-1 + Pac + Apa	33%	−
14	S-1 + Irino + Apa	49%	−
	S-1 + Pac + Apa	41%	−
	S-1 + Oxa + Apa	40%	−

^a^Irino, Irinotecan; S-1, Tegafur, Gimeracil and Oteracil Potassium Capsules; Doc, Docetaxel; Cape,Capecitabine; Oxa, Oxaliplatin;Pac, Paclitaxel; Apa, Apatinib; Cis, Cisplatin; Epi, Epirubicin; Tor, Toripalimab

^b^ T/C%$${\text{T/C}}\% ~ = \frac{{{\text{mean}}\,{\text{RLU}}\,{\text{of}}\,{\text{the}}\,{\text{treatment}}\,{\text{group}}\,{\text{on}}\,{\text{day}}\,7\,??{\text{mean}}\,{\text{RLU}}\,{\text{on}}\,{\text{day}}\,0}}{{{\text{mean}}\,{\text{RLU}}\,{\text{of}}\,{\text{the}}\,{\text{vehicle}}\,{\text{group}}\,{\text{on}}\,{\text{day}}\,7\,??\,{\text{mean}}\,{\text{RLU}}\,{\text{on}}\,{\text{day}}\,0}}$$

^c^A regimen was defined as “sensitive” when the proliferation rate of the tumor was < 50% compared to the control group

### Evaluation of short-term clinical efficacy of patients

**Fig. 2:**
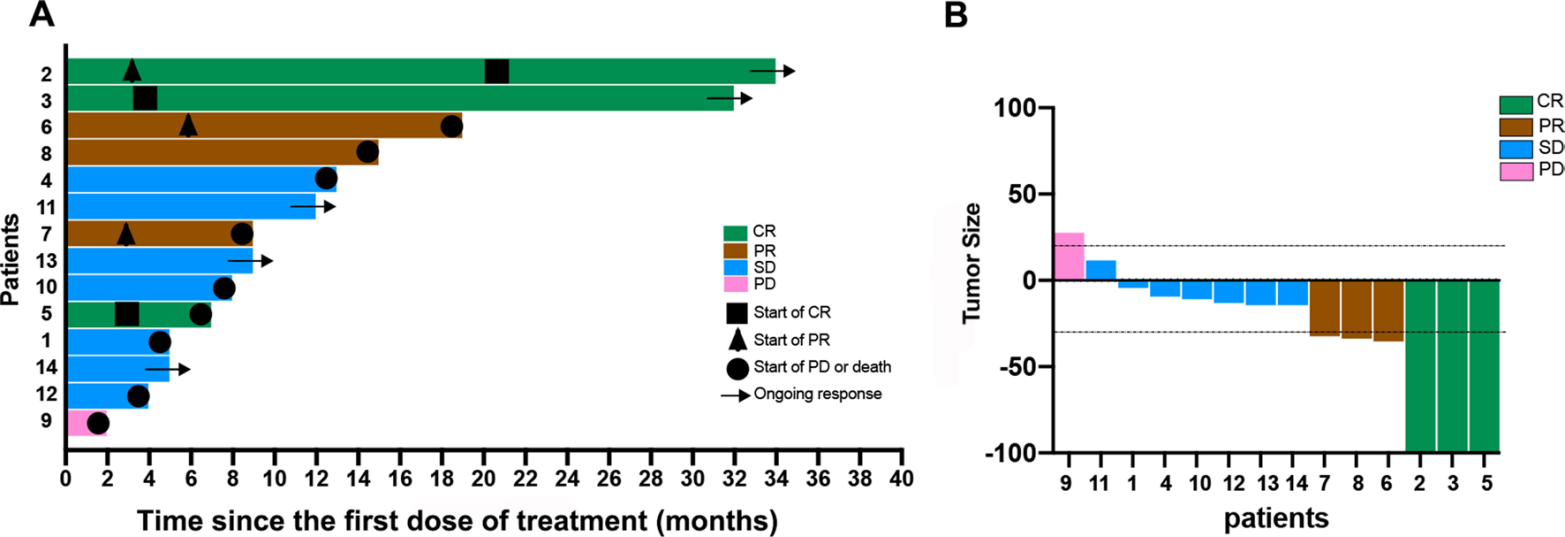
Characteristics of objective response in patients with MiniPDX drug sensitivity testing protocol. **A** Duration of response (N = 14). **B** The maximum percentage reduction from baseline in target lesions (N = 14)

According to the drug susceptibility results, a chemotherapy regimen with high sensitivity (i.e., with a low proliferation rate) was selected. The final chemotherapy protocol and corresponding efficacy evaluation showed that among the 14 patients, 3 were CR, 3 were PR, 7 were SD, and 1 was PD (Table 4). The short-term efficacy evaluation within 24 weeks showed 2 were CR, 3 were PR, 8 were SD, and 1 was PD **(**Table 5**)**. The final ORR was 42.9% (6/14, 95% CI 17.7–71.1%) and DCR was 92.9% (13/14, 95% CI 66.1–99.8%) **(**Table 6**).** DOR occurred in patient 5, 6, and 7 at 4 months, 6 months, and 13 months, respectively. Treatment was discontinued in 3 (21.4%) patients due to disease progression and in 6 (42.9%) patients due to death, with a mean duration of 6.6 ± 4.1 months. The swimming plot and waterfall plot are shown in Fig. 2.

**Table 4:** Treatment after enrollment, and clinical response

Number	Treatment regimen after drug selection^a^	Clinical outcome
1	Apatinib	SD
2	Irino + S-1	CR
3	Oxa + S-1	CR
4	S-1 + Pac	SD
5	S-1 + Doc	CR
6	S-1 + Oxa	PR
7	S-1 + Oxa	PR
8	S-1 + Oxa	PR
9	Pac + S-1 + Apa	PD
10	Cis + Cape + Apa	SD
11	Epi + Cis + 5-FU + Apa	SD
12	S-1 + Oxa + Apa	SD
13	S-1 + Oxa + Apa	SD
14	S-1 + Irino + Apa	SD

^a^Irino, Irinotecan; Doc, Docetaxel; Cape, Capecitabine; Oxa, Oxaliplatin; Pac, Paclitaxel; Apa, Apatinib; Cis, Cisplatin; Epi, Epirubicin

**Table 5:** Short-term efficacy evaluation of patients

Weeks Number	1	2	3	4	5	6	7	8	9	10	11	12	13	14
6	SD	SD	SD	SD	SD	SD	SD	SD	SD	SD	SD	SD	SD	SD
12	SD	PR	SD	SD	CR	SD	PR	SD	PD	SD	SD	SD	SD	SD
18	SD	PR	CR	SD	CR	SD	PR	SD	Death	SD	SD	Death	SD	SD
24	Death	PR	CR	SD	CR	PR	PR	SD	Death	SD	SD	Death	SD	SD

**Table 6:** Clinical efficacies of patients

	N = 14
ORR, %(95% CI)	42.9% (17.7–71.1%)
DCR, %(95% CI)	92.9% (66.1–99.8%)
BOR (Beat overall response), n (%)	
CR	3 (21.43)
PR	3 (21.43)
SD	7 (50)
PD	1 (7.14)

### Evaluation of patients’ survival

**Fig. 3:**
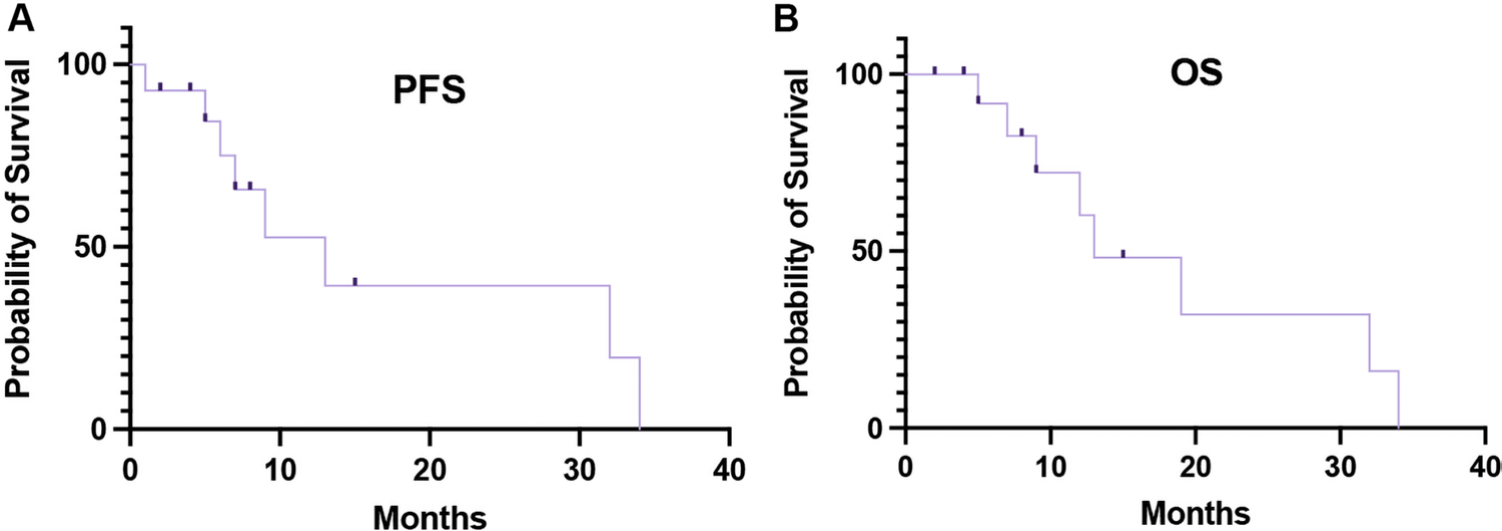
**A**, **B** Kaplan–Meier curves for progression-free survival (**A**) and overall survival (**B**) in the intent-to-treat (ITT) population

As of the data cutoff time, 6 cases of the 14 patients achieved the final outcome OS, 6 cases were alive without progression, and 2 cases died of other causes, including 1 case due to a car accident and 1 case due to a cerebrovascular accident. The median progression-free survival (mPFS) was 14.1 months (95% confidence interval: 1.18–14.82, Fig. 3A), and the mOS was 16.9 months (95% confidence interval: 2.49–23.51, Fig. 3B).

### Evaluation of patients’ quality of life

**Fig. 4:**
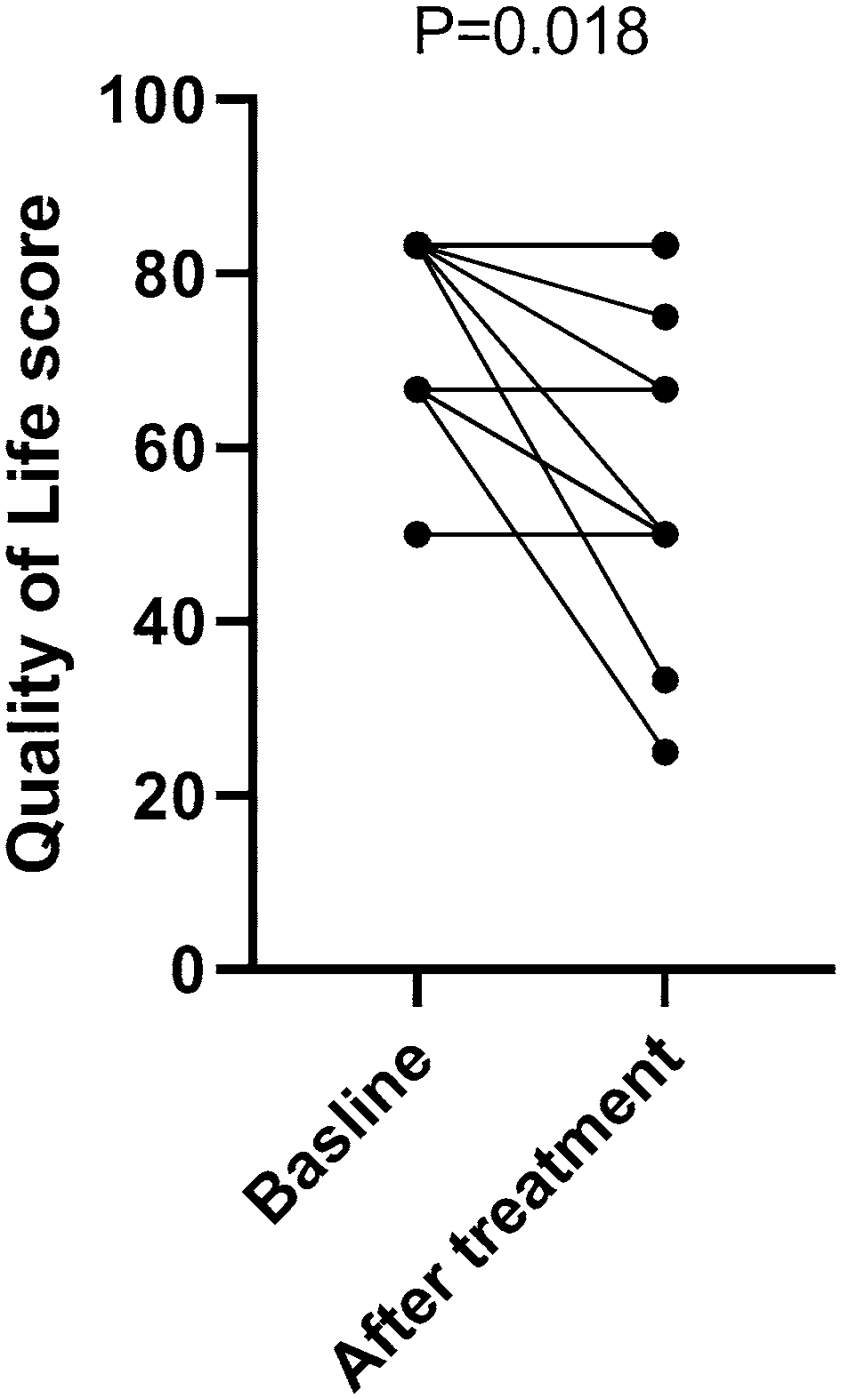
The standard score of patients’ overall quality of life before and after treatment

The overall quality of life score of 14 patients slightly decreased after treatment compared with that before treatment; the median at baseline was 75 (50–83.33) points while the median after treatment was 66.67 (25–83.33) points (P = 0.018) (Fig. 4)(Supplementary Table S1).

### Adverse reactions of patients

Fourteen patients had different degrees of adverse reactions during treatment (Table 7**)**, most of which were low-grade TRAEs, without grade 5 adverse reactions, and TRAEs were controllable. Generally, there were no new safety signals and no signs of synergistic toxicity.

**Table 7:** > 10% of patients with adverse events

Adverse event, n (%)	Any TRAEs (n = 14)	
	All grades	Grade ≥ 3
Fatigue	14 (100)	6 (42.9)
Anorexia	13 (92.9)	6 (42.9)
Insomnia	12 (85.7)	1 (7.1)
Nausea	11 (78.6)	2 (14.3)
Anhelation	10 (71.4)	1 (7.1)
Pain	8 (57.1)	1 (7.1)
Hepatic insufficiency	8 (57.1)	2 (14.3)
Leukopenia	5 (35.7)	3 (21.4)
Anemia	5 (35.7)	3 (21.4)
Constipation	4 (28.6)	2 (14.3)
Platelet count decreased	4 (28.6)	2 (14.3)

## Discussion

Cancer is a highly heterogeneous disease affected by genomic, epigenetic, and transcriptome changes in cells. Even tumors originating from the same organ have differences, so personalized medicine or personalized care is crucial when designing the treatment plan [21]. Over the years, personalized therapies based on genetic test results of GC/GJC patients have been increasing. In this study, three different combined chemotherapy regimens were selected for different GC/GJC patients according to NCCN guidelines (2016 edition) [19]. The sensitivity of these three combined chemotherapy regimens was analyzed using a MiniPDX model, and the chemotherapy regimen with the lowest proliferation rate was selected for individualized treatment of patients according to the detection results. The results showed that ORR was 42.86% (6/14) and DCR was 92.86% (13/14); the mean PFS time was 14.1 months (95% CI 1.18 -14.82), and the mean OS time was 16.9 months (95% CI 2.49–23.51). The overall quality of life score was slightly lower after treatment than before treatment, and the occurrence rate of grade 3–4 chemotherapy adverse reactions such as bone marrow suppression, nausea and vomiting, and abnormal liver enzymes was relatively low. Therefore, the MiniPDX-guided treatment was found to have better safety and better survival time.

Fluorouracil plus platinum-based chemotherapy is the most commonly used first-line therapy for unresectable advanced or metastatic human epidermal growth factor receptor 2 (HER2)-negative gastric and gastroesophageal junction adenocarcinoma, with a median overall survival rate (OS) less than 1 year [22–24]. However, the clinical benefit rates of second-line chemotherapy, including SPA (S-1 and paclitaxel) [24], XELOX (capecitabine and oxaliplatin) [25, 26], DOCOX (docetaxel plus oxaliplatin) [27], S-1 monotherapy, XELIRI (capecitabine and irinotecan) [28] and some newly developed targeted agents (such as apatinib monotherapy) [29], were even more limited. At present, no large randomized controlled clinical trial has been able to prove that maintenance chemotherapy can improve patient survival time. Although new targeted therapies provide more possibilities for maintenance therapy strategies, most completed clinical trials failed to achieve OS prolongation [8].In our study, one patient was reported to have an EGFR mutation by genetic testing, and this overexpression was associated with poor prognosis [30]. The combination of anti-EGFR therapy with chemotherapy provides a basis for the treatment of resistant GC patients.Several phase II clinical trials demonstrated the benefit of combination chemotherapy and anti-EGFR therapy in GC patients [31–33], so we tried drug susceptibility testing with AZD9291, but the results were not satisfactory.

The patient-derived xenograft (PDX) model is commonly used for in vivo experiments, as it can reflect the molecular diversity and cellular and histological heterogeneity of tumors in patients [34–36]. However, the long trial period and unsatisfactory implantation rate hinder the widespread application of PDX in some advanced malignancies, especially in GC [37]. In recent years, the clinical application of MiniPDX has become more and more common, and encouraging positive results have been obtained in the application of hepatocellular carcinoma [38] and gallbladder carcinoma [39]. There were also different potential applications in other solid tumors. For example, in another case reported by Zhao et al. [40], personalized treatment based on MiniPDX and whole-exome sequencing was used to rapidly assess drug sensitivity and reveal significant genetic changes in patients with metastatic duodenal adenocarcinoma. Moreover, Liu et al. [41] found that gemcitabine and XCT790 have synergistic antitumor effects on pancreatic cancer by the MiniPDX model. By establishing the MiniPDX model, Xu et al. [42] found that the combined application of AKT inhibitors and PARP inhibitors might be a feasible method for clinical trials in patients with recurrent ovarian cancer. As mentioned above, the research of MiniPDX in advanced gastric cancer is still limited to retrospective studies and case reports. Our prospective study revealed a longer median PFS time and median OS time, thus providing more convincing evidence for the feasibility of MiniPDX in HER2-negative advanced gastric cancer.

However, the drug susceptibility testing technology of MiniPDX also has certain limitations. First, the effects of chemotherapy, which worked by regulating the cell cycle, might not be adequately reproduced because the MiniPDX model was used within 7 days, so the MiniPDX model could not simulate the actual administration and effects of the entire cycle. Second, based on the Checkmate649 study [43], immunotherapy had been included in the first-line treatment of advanced gastric cancer. Since our study was carried out in 2018 and immunotherapy was not included in the latest guidelines at that time, the PD-L1 expression rate of patients was not detected, and immune drugs were not included in the drug sensitivity test. Moreover, the nude mice used in this study could not reproduce the human immune environment, meaning that the efficacy of combined immunotherapy could not be tested. Yet, a humanized peripheral blood mononuclear cell (PBMC) reconstruction model of immunodeficient mice could be used to evaluate the efficacy of immunotherapy drugs, and drug sensitivity testing programs can be extended to guide clinical medication in the future. Other shortcomings are a lack of multi-center, multi-sample size, and control group. In the future, we plan to further expand the sample size and summarize more clinical data to obtain a higher-level evidence-based basis.

In conclusion, the MiniPDX model has shown to be an effective tool to guide the choice of drug regimens for GC/GJC patients, providing a scientific basis for clinical conversion therapy of GC/GJC, reducing adverse reactions to clinical experience-guided medication, and revealing broad application prospects. In the future, randomized controlled trials with larger sample sizes are needed to further clarify the efficacy and safety.

## Supplementary Information

Additional file1 (DOCX 21 KB)

## Data Availability

The datasets generated during and/or analysed during the current study are available from the corresponding author on reasonable request.
