# Supplementary material for: OncoVee™-MiniPDX-guided anticancer treatment for HER2-negative intermediate-advanced gastric cancer patients: a single-arm, open-label phase I clinical study
Source: Discov Oncol. 2023 Apr 24;14:46. doi: 10.1007/s12672-023-00661-y (PMC10126180; doi:10.1007/s12672-023-00661-y)
Supplement: Supplementary file 1 — Additional file1 (DOCX 21 KB) [file 12672_2023_661_MOESM1_ESM.docx]

**Supplementary materials**

Comparison of QLQ-C30 scores of patients before and after treatment

| Number | Pre-treatment physical function | Post-treatment physical function | Pre-treatment role function | Post-treatment role function | Pre-treatment emotional function | Post-treatment emotional function | Pre-treatment cognitive function | Post-treatment cognitive function | Pre-treatment social function | Post-treatment social function | Pre-treatment fatigue | Post-treatment fatigue | Pre-treatment nausea and vomiting | Post-treatment nausea and vomiting | Pre-treatment pain | Post-treatment pain | Pre-treatment shortness of breath | Post-treatment shortness of breath | Pre-treatment insomnia | Post-treatment insomnia | Pre-treatment appetite loss | Post-treatment appetite loss | Pre-treatment constipation | Post-treatment constipation | Pre-treatment diarrhea | Post-treatment diarrhea | Pre-treatment financial hardship | Post-treatment financial hardship |
| --- | --- | --- | --- | --- | --- | --- | --- | --- | --- | --- | --- | --- | --- | --- | --- | --- | --- | --- | --- | --- | --- | --- | --- | --- | --- | --- | --- | --- |
| 1 | 80 | 66.67 | 66.67 | 66.67 | 100 | 100 | 83.33 | 83.33 | 66.67 | 66.67 | 33.33 | 44.44 | 0 | 16.67 | 33.33 | 33.33 | 33.33 | 66.67 | 0 | 0 | 33.33 | 66.67 | 0 | 0 | 0 | 0 | 0 | 0 |
| 2 | 100 | 93.33 | 100 | 100 | 100 | 100 | 100 | 100 | 100 | 100 | 22.22 | 22.22 | 0 | 0 | 0 | 0 | 0 | 0 | 0 | 33.33 | 33.33 | 33.33 | 0 | 0 | 0 | 0 | 0 | 0 |
| 3 | 100 | 93.33 | 100 | 83.33 | 100 | 100 | 100 | 100 | 83.33 | 83.33 | 33.33 | 33.33 | 16.67 | 16.67 | 0 | 0 | 33.33 | 33.33 | 33.33 | 33.33 | 33.33 | 33.33 | 0 | 0 | 0 | 0 | 0 | 0 |
| 4 | 86.67 | 86.67 | 83.33 | 100 | 91.67 | 100 | 100 | 100 | 83.33 | 66.67 | 33.33 | 33.33 | 0 | 0 | 0 | 0 | 33.33 | 33.33 | 33.33 | 33.33 | 33.33 | 33.33 | 33.33 | 66.67 | 0 | 0 | 0 | 0 |
| 5 | 66.67 | 66.67 | 50 | 50 | 100 | 100 | 83.33 | 100 | 66.67 | 66.67 | 55.56 | 55.56 | 33.33 | 33.33 | 0 | 16.67 | 0 | 0 | 33.33 | 33.33 | 33.33 | 33.33 | 33.33 | 33.33 | 0 | 0 | 0 | 33.33 |
| 6 | 100 | 100 | 100 | 100 | 100 | 100 | 100 | 100 | 83.33 | 83.33 | 33.33 | 33.33 | 16.67 | 16.67 | 0 | 0 | 0 | 0 | 33.33 | 33.33 | 33.33 | 33.33 | 0 | 0 | 66.67 | 0 | 0 | 0 |
| 7 | 100 | 100 | 83.33 | 83.33 | 91.67 | 91.67 | 100 | 100 | 66.67 | 66.67 | 33.33 | 33.33 | 0 | 16.67 | 0 | 0 | 0 | 0 | 33.33 | 33.33 | 33.33 | 33.33 | 33.33 | 33.33 | 0 | 0 | 0 | 0 |
| 8 | 93.33 | 60 | 100 | 100 | 100 | 100 | 100 | 100 | 83.33 | 83.33 | 33.33 | 33.33 | 16.67 | 16.67 | 33.33 | 33.33 | 0 | 0 | 33.33 | 33.33 | 33.33 | 33.33 | 0 | 0 | 0 | 0 | 0 | 0 |
| 9 | 66.67 | 40 | 66.67 | 33.33 | 91.67 | 91.67 | 83.33 | 83.33 | 66.67 | 66.67 | 55.56 | 66.67 | 50 | 100 | 33.33 | 33.33 | 0 | 33.33 | 33.33 | 33.33 | 66.67 | 100 | 0 | 0 | 0 | 0 | 0 | 0 |
| 10 | 100 | 66.67 | 100 | 66.67 | 83.33 | 91.67 | 100 | 83.33 | 83.33 | 83.33 | 33.33 | 33.33 | 0 | 0 | 33.33 | 33.33 | 0 | 0 | 33.33 | 33.33 | 33.33 | 33.33 | 33.33 | 33.33 | 0 | 0 | 0 | 0 |
| 11 | 66.67 | 60 | 66.67 | 66.67 | 83.33 | 75.00 | 66.67 | 66.67 | 66.67 | 66.67 | 33.33 | 33.33 | 0 | 0 | 0 | 0 | 33.33 | 33.33 | 33.33 | 33.33 | 33.33 | 0 | 0 | 0 | 0 | 0 | 0 | 0 |
| 12 | 80 | 80 | 66.67 | 66.67 | 100 | 100 | 100 | 100 | 83.33 | 83.33 | 33.33 | 33.33 | 0 | 0 | 33.33 | 33.33 | 33.33 | 33.33 | 33.33 | 33.33 | 33.33 | 33.33 | 0 | 0 | 0 | 0 | 0 | 0 |
| 13 | 66.67 | 66.67 | 66.67 | 66.67 | 91.67 | 83.33 | 100 | 100 | 83.33 | 83.33 | 33.33 | 33.33 | 33.33 | 0 | 0 | 0 | 0 | 0 | 33.33 | 33.33 | 0 | 0 | 0 | 0 | 0 | 0 | 0 | 0 |
| 14 | 93.33 | 93.33 | 83.33 | 83.33 | 100 | 100 | 100 | 100 | 66.67 | 66.67 | 22.22 | 33.33 | 0 | 0 | 0 | 0 | 0 | 0 | 0 | 0 | 0 | 0 | 0 | 0 | 0 | 0 | 0 | 0 |

Evaluation of patients after treatment

| Number | Time of first treatment | Survival or not* | Time of death | OS(months) | PFS(months) | Time to PD | Time to PR | Time to CR | ORR | Target lesion location | Baseline value(mm) | Final value(mm) | Change |
| --- | --- | --- | --- | --- | --- | --- | --- | --- | --- | --- | --- | --- | --- |
| 1 | 2018.08.23 | 0 | 2019.01.20 | 5 | 5 | Reach OS |  |  | SD | Left lobe of liver | 52 | 53 | +2% |
| 2 | 2019.02.11 | 1 |  | 34 | 34 | None | 2019.05 | 2020.08 | CR | Lower esophageal segment | 31 | 0 | -100% |
| 3 | 2019.04.17 | 1 |  | 32 | 32 | None |  | 2019.08 | CR | Mediastinal lymph nodes | 18 | 0 | -100% |
| 4 | 2019.06.27 | 1(Death from other diseases) | 2020.07.01 | 13 | 13 | Reach OS |  |  | SD | Left lobe of liver | 33 | 29 | -12% |
| 5 | 2020.02.26 | 1(Death from other diseases) | 2020.09.01 | 7 | 7 | Reach OS |  | 2020.05 | CR | Perigastric lymph nodes | 20 | 0 | -100% |
| 6 | 2020.05.14 | 0 | 2021.12.25 | 19 | 6 | Reach OS | 2021.01 |  | PR | Right lobe of liver | 20 | 13 | -35% |
| 7 | 2020.05.23 | 0 | 2021.02.01 | 9 | 7 | 2020.12.10 | 2020.08 |  | PR | Both lungs and right kidney | 43 | 29 | -33% |
| 8 | 2020.05.29 | 0 | 2021.07.13 | 15 | 12 | 2021.05.01 |  |  | PR | Intrahepatic parenchyma | 40 | 26 | -35% |
| 9 | 2020.09.21 | 0 | 2020.11.02 | 2 | 2 | Reach OS |  |  | PD | Left adrenal gland, perigastric lymph nodes, abdominal and retroperitoneal lymph nodes | 20 | 26 | +30% |
| 10 | 2020.10.03 | 0 | 2021.05.15 | 7 | 7 | 2021.05.15 |  |  | SD | Liver | 31 | 27 | -13% |
| 11 | 2020.12.09 | 1 |  | 12 | 1 | 2021.01.27 |  |  | SD | Right ovary | 66 | 70 | +5% |
| 12 | 2020.12.28 | 0 | 2021.04.21 | 4 | 4 | Reach OS |  |  | SD | Right lobe of liver | 38 | 33 | -13% |
| 13 | 2020.03.19 | 1 |  | 9 | 9 | None |  |  | SD | Mediastinal lymph nodes | 20 | 17 | -15% |
| 14 | 2020.07.30 | 1 |  | 5 | 5 | None |  |  | SD | Anastomotic opening | 28 | 24 | -14% |

*1：survival;0:death
